# Characterization of a Conjugative Multidrug Resistance IncP-2 Megaplasmid, pPAG5, from a Clinical Pseudomonas aeruginosa Isolate

**DOI:** 10.1128/spectrum.01992-21

**Published:** 2022-02-16

**Authors:** Meng Li, Congcong Guan, Gaoyu Song, Xiaoxi Gao, Weina Yang, Tietao Wang, Yani Zhang

**Affiliations:** a Key Laboratory of Resource Biology and Biotechnology in Western China, Ministry of Education, Northwest University, Xi’an, People’s Republic of China; b Provincial Key Laboratory of Biotechnology of Shaanxi Province, Northwest University, Xi’an, People’s Republic of China; c Department of Clinical Laboratory, The Children’s Hospital of Xi’an City, Xi’an, People’s Republic of China; University of Guelph

**Keywords:** *Pseudomonas aeruginosa*, conjugative plasmid, multidrug resistance, IncP-2 plasmid, plasmid evolution

## Abstract

The spread of resistance genes via horizontal plasmid transfer plays a significant role in the formation of multidrug-resistant (MDR) Pseudomonas aeruginosa strains. Here, we identified a megaplasmid (ca. 513 kb), designated pPAG5, which was recovered from a clinical multidrug-resistant P. aeruginosa PAG5 strain. The pPAG5 plasmid belonged to the IncP-2 incompatibility group. Two large multidrug resistance regions (MDR-1 and MDR-2) and two heavy metal resistance operons (*merEDACPTR* and *terZABCDE*) were identified in the pPAG5 plasmid. Genetic analysis demonstrated that the formation of MDR regions was mediated by several homologous recombination events. Further conjugation assays identified that pPAG5 could be transferred to P. aeruginosa but not Escherichia coli. Antimicrobial susceptibility testing on transconjugants demonstrated that pPAG5 was capable of transferring resistance genes to transconjugants and producing a multidrug-resistant phenotype. Comparative analysis revealed that pPAG5 and related plasmids shared an overall similar backbone, including genes essential for replication (*repA*), partition (*par*), and conjugal transfer (*tra*). Further phylogenetic analysis showed that pPAG5 was closely related to plasmids pOZ176 and pJB37, both of which are members of the IncP-2-type plasmid group.

**IMPORTANCE** The emergence and spread of plasmid-associated multidrug resistance in bacterial pathogens is a key global threat to public health. It is important to understand the mechanisms of the formation and evolution of these plasmids in patients, hospitals, and the environment. In this study, we detailed the genetic characteristics of a multidrug resistance IncP-2 megaplasmid, pPAG5, and investigated the formation of its MDR regions and evolution. To the best of our knowledge, plasmid pPAG5 is the largest multidrug resistance plasmid ever sequenced in the Pseudomonas genus. Our results may provide further insight into the formation of multidrug resistance plasmids in bacteria and the molecular evolution of plasmids.

## INTRODUCTION

The presence of multidrug-resistant (MDR) pathogens is one of the most important global public health threats (1). Pseudomonas aeruginosa is an opportunistic pathogen and leading cause of nosocomial infections, which are often difficult to eradicate due to multidrug resistance (2). The mechanisms underlying antimicrobial resistance (AMR) in P. aeruginosa may be intrinsic to the species or acquired through mutation of intrinsic genes or horizontal gene transfer from other bacteria that carry genetic material encoding resistance determinants (3).

Whole-genome sequencing suggests that the spread of resistance genes via horizontal plasmid transfer plays a significant role in determining P. aeruginosa AMR (4). Most of the identified transmissible resistance plasmids in P. aeruginosa belong to the IncP-2 incompatibility group (5). IncP-2 plasmids often possess several typical characteristics (5–7). First, they may have a narrow host range, as IncP-2 plasmids have not been transferred to E. coli from P. aeruginosa by conjugation. Second, they are usually single copy and have a large size (8). Third, IncP-2 plasmids are resistant to metals (tellurium, mercury, and chromate) and possess bacteriophage-inhibiting properties. Recent reports also revealed that IncP-2 plasmids like pJB37, pOZ176, pBT2436, and pBT2101 carry multiple MDR cassettes (2, 5, 6). These characteristics may provide adaptive advantages for their host, especially in hospital settings. However, the step-by-step evolutionary changes in these megaplasmids remain unknown.

Here, we report the largest multidrug resistance plasmid ever sequenced in the Pseudomonas genus, pPAG5. In addition, we identify the detailed genetic characteristics of the multidrug resistance IncP-2 megaplasmid pPAG5 and investigate the formation of MDR regions and evolution of pPAG5. Our results may provide further insight into the formation of multidrug resistance plasmids in bacteria and the genomic diversity and molecular evolution of plasmids.

## RESULTS

### General features of the pPAG5 plasmid.

A previous molecular epidemiologic study used whole-genome sequencing to identify a clinical P. aeruginosa PAG5 isolate that contained the megaplasmid pPAG5 (9). However, pPAG5 was not yet characterized. To do this, its genomic backbone was examined in detail. The complete plasmid sequence of pPAG5 was 513,322 bp long and had a guanine-cytosine (GC) content of 56.31%. Plasmid pPAG5 was annotated using the NCBI Prokaryotic Genome Annotation Pipeline (PGAP) and the RAST server. It contained 538 protein-coding sequences (CDS), 57.7% of which encoded hypothetical proteins. The pPAG5 plasmid backbone contains key genes of IncP-2-type plasmids, including replication (*repA*), partitioning (*parA* and *parB*), and transfer (*traI*, *traG*, and *virB4*) genes. Virulence factors were identified in the pPAG5 plasmid, including twitching motility genes (*pilB*, *pilG*, *pilT*, and *pilZ*), a chemotaxis operon (*cheBARZWY*), and virulence regulatory genes (*vfr* and *csrA*). Based on the read coverage versus chromosome sequencing data, pPAG5 was present as a single-copy plasmid.

A BLASTp comparison against the GenBank database using the full plasmid sequence showed that the majority of CDS from the pPAG5 plasmid were similar to those of plasmid pOZ176 in P. aeruginosa strain PA96, isolated from a clinical specimen (6). Plasmid pOZ176 was categorized as a member of incompatibility group IncP-2 by phenotypic methods (6). A pairwise comparison of pPAG5 and pOZ176 indicated that these two plasmids encode highly similar RepA replication proteins and ParA and ParB partition proteins (100%, 100%, and 99% amino acid sequence identities, respectively), suggesting that pPAG5 belonged to the IncP-2 group.

### Two MDR regions in plasmid pPAG5.

AMR genes were detected through BLASTn searches against the Comprehensive Antibiotic Resistance Database (CARD) (10). Structure alignments of MDR regions were compared using the Easyfig tool (11). Two concentrated AMR regions were identified in pPAG5 and designated MDR-1 and MDR-2 (25.3 kb and 7.9 kb in length, respectively). Both resistance regions were rich in transposases and integrase. Five different insertion sequences (ISs) were identified as scattered across MDR-1, whereas one IS was identified in MDR-2. The origins of the ISs were diverse and included Pseudomonas alcaligenes, Escherichia coli, Aeromonas salmonicida, and Proteus vulgaris. This indicated that ISs play a key role in the acquisition of resistance from different sources.

**FIG 1:**
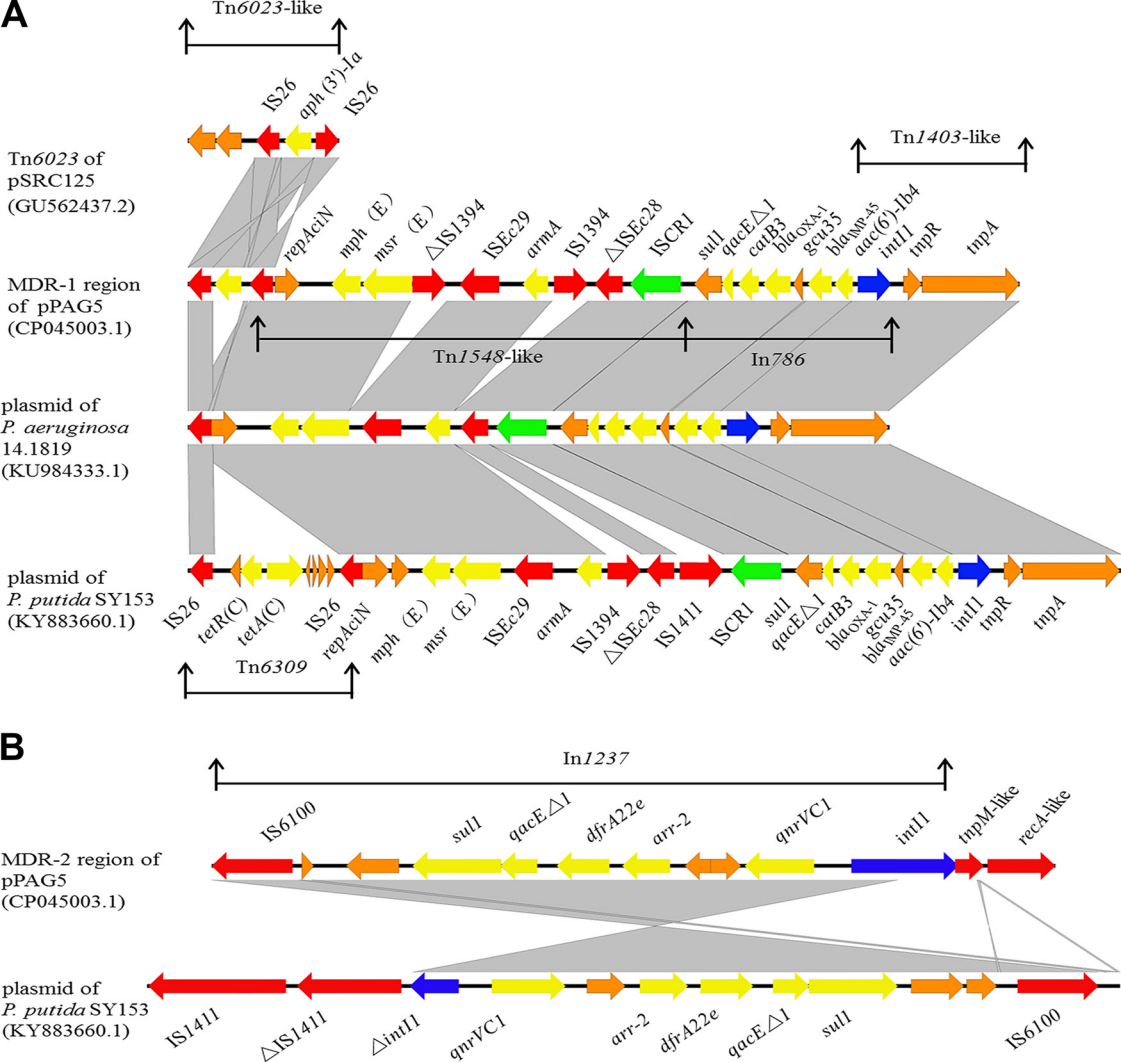
Structure alignment of various plasmid carrying the MDR-1 region or MDR-2 region, and comparison of plasmids in identical gene environments. (A) Alignment of pPAG5 and plasmids carrying a similar MDR-1 region using Easyfig. (B) Alignment of pPAG5 and plasmids carrying a similar MDR-2 region using Easyfig. Antibiotic resistance genes are shown by yellow arrows, insertion sequences(ISs) by red arrows, IS common region 1 by green arrows, and class 1 integrons by blue arrows. Orange arrows indicate other functional genes. Regions of homology are marked by gray shading.

The MDR-1 region was associated with integron In*786*, transposon Tn*1403*-like, Tn*1548*-like, and Tn*6023*-like (Fig. 1A). Integron In*786*, with MDR gene cassette array *aac*(*6′*)*-Ib4 -bla*_IMP-45_-*gcu3-bla*_OXA-1_-*catB3*, contributes resistance to aminoglycosides, β-lactams, and chloramphenicol. Upstream from In*786*, a Tn*1403*-like transposon containing *tnpA* and *tnpR* was also identified and was 99% identical to the amino acid sequence of Tn*1403.* Downstream from In*786*, a Tn*1548*-like transposon carried the resistance module *armA-msr(E)-mph(E)*-*repAciN*, encoding high-level resistance to aminoglycosides and macrolides. Upstream from this module, IS*1394* was flanked by IS*CR1*. IS*CR1* elements can transfer adjacent DNA sequences into other bacterial species (12). Downstream from the module, there was a Tn*6023* transposon carrying an *aph(3′)*-*Ia* gene flanked by two IS*26* insertion sequences, contributing resistance to aminoglycosides (13). The MDR-1 region demonstrated a high similarity to the MDR region of plasmid pPA1819 in P. aeruginosa strain 14.1819 (87% coverage and 99.98% identity) and that of plasmid pSY153-MDR in Pseudomonas putida strain SY153 (96% coverage and 99.99% identity) (Fig. 1A).

The MDR-2 region consisted of In*1237*, *tnpM*-like, and *recA*-like (Fig. 1B). Integron In*1237* was flanked upstream by IS*6100* and downstream by *tnpM*-like and *recA*-like. In*1237* contained the *qnrVC1-gcu165-arr-2-dfrA22e* cassette array, which confers resistance to quinolones, rifampicin, and dihydrofolate. Of note, this cassette was first reported in pPA1819 from P. aeruginosa strain 14.1819 (14). The same array also has been observed previously in pSY153-MDR from P. putida strain SY153 (15). The TnpM-like transposase shared 95% amino acid identity with the TnpM transposase of plasmid pNDM-CIT, which belonged to the IncHI1 plasmid type found in the extremely drug-resistant Citrobacter freundii strain STE (16). The RecA-like recombinase displayed 85% amino acid similarity to the RecA protein of plasmid pMBUI7, which belongs to the self-transmissible plasmid and IncU plasmid type, carried by an uncultured bacterium isolated from Paradise Creek (17). The MDR-2 region was very similar to the MDR region of plasmid pSY153-MDR from P. putida SY153 (94% coverage and 99.98% identity) (Fig. 1B).

### Characterization of heavy metal resistance operons.

**FIG 2:**
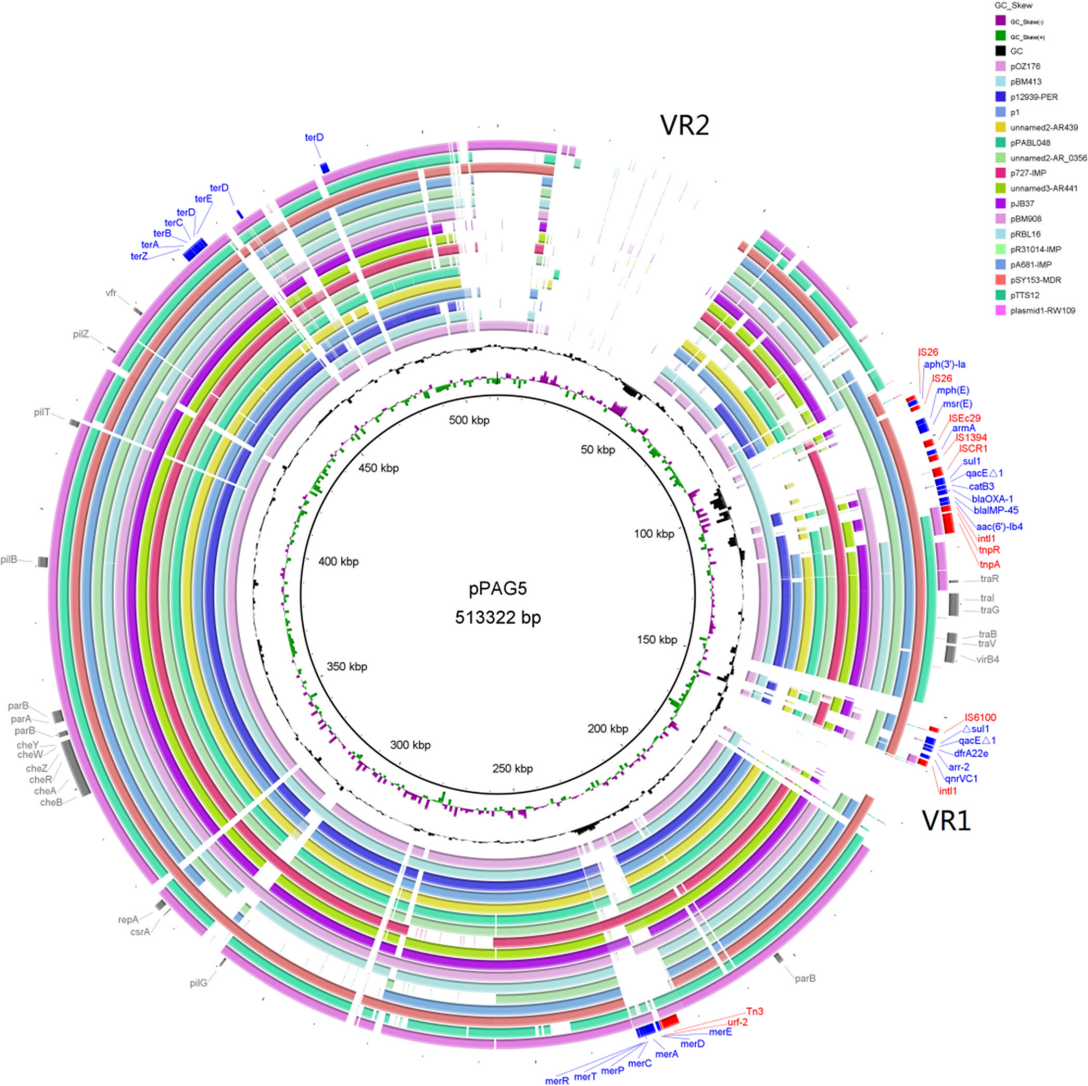
Genome comparison of pPAG5 and related megaplasmids. Seventeen complete Pseudomonas megaplasmid sequences are identified by colored rings as shown in the key at the top right. These were aligned to the pPAG5 genome at the nucleotide level. Solid ring segments denote sequence homology to pPAG5, whereas gaps within the rings correspond to regions lacking sequence similarity. The scale is indicated on the innermost ring. The second ring illustrates guanine-cytosine (GC) skew. The next ring represents the GC content deviation from the average in reference genomes. The outermost ring indicates the locations of genes encoding key features (gray), genes encoding integrases or transposases (red), and AMR genes (blue) in the pPAG5 plasmid. The genome comparison map was generated using BRIG software (version 0.95).

Two heavy metal resistance operons, encoding resistance to tellurite and mercury, were observed in pPAG5 (Fig. 2). The tellurite operon consisted of *terZABCDE* genes and two genes encoding members of the TerD family of proteins. This tellurite operon exhibited 100% amino acid identity with the tellurite operon of plasmid pOZ176 (6). Resistance to tellurite is a key feature of IncP-2 Pseudomonas plasmids; in fact, they all carry resistance to tellurite. The mercury operon contained *merEDACPTR* genes and was 100% identical to the mercury operon of plasmid p727-IMP (18). Of the 17 pPAG5-related plasmids, only 9 plasmids possess the mercury operon, which can provide resistance to mercury (Fig. 2).

### Transmissibility of pPAG5.

**FIG 3:**
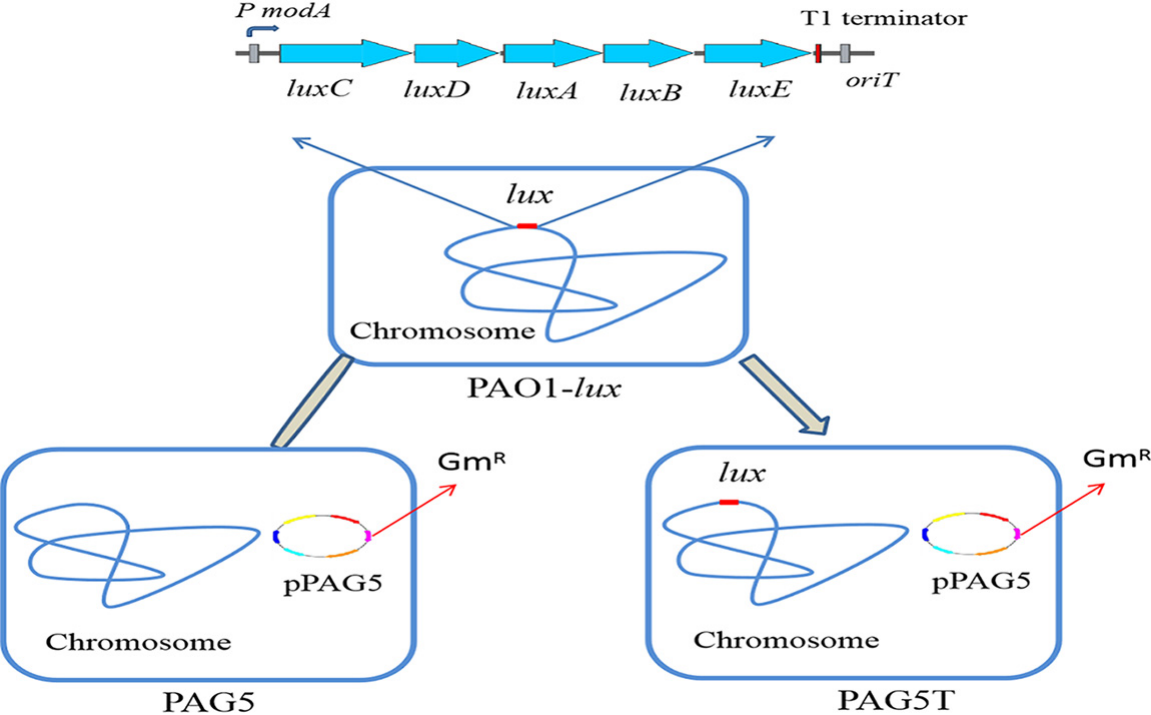
Schematic diagram of conjugal transfer of pPAG5 from the donor strain P. aeruginosa PAG5 to the recipient strain P. aeruginosa PAO1-*lux*. The conjugative donor strain PAG5 contains plasmid pPAG5 (small circle). The recipient strain PAO1-*lux* contains a chromosomally integrated *luxCDABE* operon (red). The transconjugant strain PAG5T contains plasmid pPAG5 and the *luxCDABE* operon. Gm^R^, gentamicin resistance gene.

The annotation of plasmid pPAG5 demonstrated that it contained *oriT*, *virB4*, and *traG* genes required for conjugative transfer (19). Therefore, conjugation experiments were performed to determine whether pPAG5 would undergo intra- and interspecies horizontal transfer. The donor strain PAG5 and recipient strain PAO1-*lux* were subjected to plasmid transfer experiments. Plasmid pPAG5 encoded high-level resistance to gentamycin (Gm) (Fig. 3), and therefore, the recipient strain PAO1-*lux* would acquire Gm resistance if pPAG5 was successfully transferred into it. Mating between PAG5 and PAO1-*lux* in LB for 24 h resulted in the appearance of Gm-resistant bioluminescent transconjugants (Fig. 3). However, it failed to transfer the plasmid to E. coli strain DH5α-*lux*. This finding showed that pPAG5 is self-transmissible but has a narrow host range.

### Multidrug resistance phenotype.

The MICs of amikacin, aztreonam, ceftazidime, ciprofloxacin, colistin, cefepime, gentamicin, imipenem, levofloxacin, meropenem, piperacillin, and piperacillin-tazobactam were determined for PAG5, the transconjugant strain PAG5T, and PAO1-*lux* by conducting broth dilution tests. PAG5 showed resistance to all antibiotics except colistin. The transconjugant PAG5T exhibited resistance to all antibiotics except aztreonam, colistin, and imipenem. However, PAO1-*lux* was susceptible to all antibiotics. The transconjugant PAG5T and donor PAG5 had similar patterns of MICs to meropenem (MIC > 8 μg/mL), ceftazidime (MIC > 16 μg/mL), piperacillin (MIC > 64 μg/mL), cefepime (MIC > 16 μg/mL), gentamicin (MIC > 8 μg/mL), piperacillin-tazobactam (MIC > 64 μg/mL), and amikacin (MIC > 8 μg/mL) (Table 1). These results indicated that pPAG5 could transfer these associated antibiotic resistance genes from the donor strain PAG5 to the recipient strain PAO1-*lux*, resulting in transconjugant PAG5T exhibiting a resistant phenotype.

**TABLE 1:** Antimicrobial susceptibility patterns of P. aeruginosa strain PAG5, its transconjugant strain PAG5T, and the recipient strain P. aeruginosa PAO1-*lux*

P. aeruginosa strain	MIC (mg/L) of^*a*^:											
	IPM	MEM	CAZ	PIP	FEP	CIP	LEV	GEN	TZP	ATM	AMK	CST
PAG5	>8	>8	>16	>64	>16	>2	>8	>8	>64	>16	>32	1
PAG5T	4	>8	>16	>64	>16	2	4	>8	>64	8	>32	1
PAO1-*lux*	2	≤1	2	≤4	4	≤0.5	≤1	≤2	≤4	8	≤8	1

a AMK, amikacin; ATM, aztreonam; CAZ, ceftazidime; CIP, ciprofloxacin; CST, colistin; FEP, cefepime; GEN, gentamicin; IPM, imipenem; LVX, levofloxacin; MEM, meropenem; PIP, piperacillin; TZP, piperacillin-tazobactam.

### Comparative analysis between pPAG5 and related megaplasmids.

**FIG 4:**
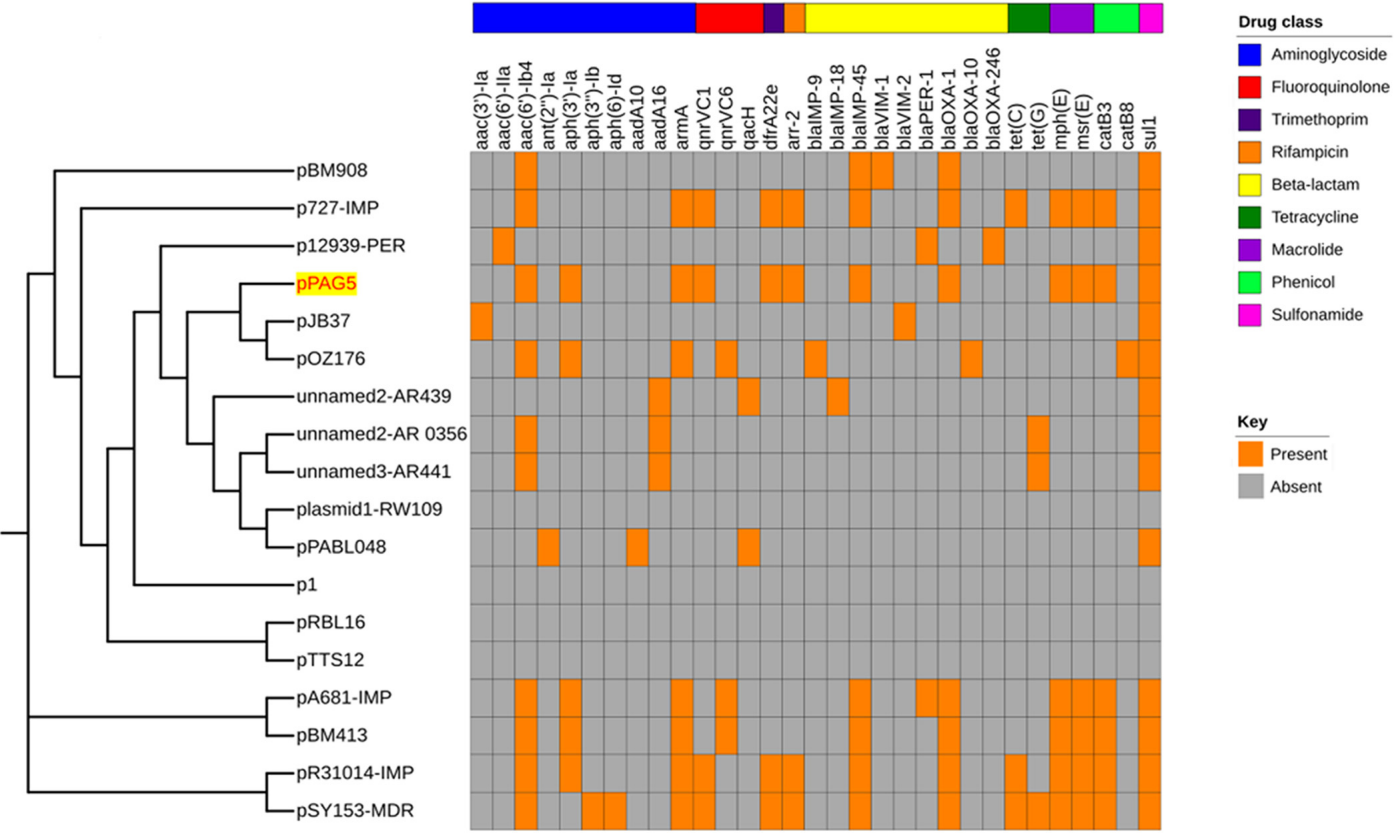
Antimicrobial resistance (AMR) gene content of pPAG5 and related megaplasmids. A phylogenetic tree displaying the relationships of the megaplasmid pPAG5 with its 17 best hits in GenBank is shown. A present/absent heatmap shows AMR gene content according to the Comprehensive Antibiotic Resistance Database (CARD). Orange displays the presence and gray shows the absence of an AMR gene. AMR genes are classified based on the drug class they confer resistance to according to CARD. The clade containing the reference pPAG5 plasmid is highlighted (yellow background and red font).

We carried out homology searches targeting pPAG5 complete sequences using the NCBI nonredundant nucleotide database. The resistance determinants were identified using ResFinder (20) and RGI software from the CARD database (10). Details of the 17 additional megaplasmids identified, including the size range and GC content, are shown in Table 2. Thirteen of 17 megaplasmids were present in strains of P. aeruginosa, and 4 related plasmids were identified in non-*aeruginosa* Pseudomonas species (Pseudomonas koreensis, Pseudomonas citronellolis, and Pseudomonas putida). Nevertheless, four of the related megaplasmids lacked any AMR genes: p1 (GenBank accession number CP027478.1), pTTS12 (GenBank accession number CP009975.1), pRBL16 (GenBank accession number CP015879.1), and plasmid 1-RW109 (GenBank accession number LT969519.1) (Fig. 4). These four megaplasmids were from the environmental isolate strain P. koreensis P19E3, soil strain P. putida S12, sludge strain P. citronellolis SJTE-3, and industrial strain P. aeruginosa RW109, respectively. Other related megaplasmids were sourced from clinical strains, with the majority belonging to P. aeruginosa.

**TABLE 2:** Megaplasmids identified by BLASTn search with pPAG5

Plasmid	% GC content	Score		Query coverage (%)	E value	% identity	Species	Size (bp)	Yr of isolation	Source	Country	Genbank accession no.
		Maximum	Total									
pOZ176	57.60	1.75E+05	7.17E+05	78	0	98.97	P. aeruginosa	500839	2000	Clinical	China	KC543497.1
pBM413	56.41	1.58E+05	7.82E+05	79	0	99.69	P. aeruginosa	423017	2012	Clinical	China	CP016215.1
p12939-PER	57.30	1.58E+05	6.47E+05	70	0	99.61	P. aeruginosa	496436	NA^*a*^	Clinical	China	MF344569.1
p1	55.97	1.57E+05	6.49E+05	73	0	99.47	P. koreensis	467568	2014	Environment	Switzerland	NZ_CP027478.1
Unnamed2-AR439	56.87	1.55E+05	6.52E+05	73	0	98.81	P. aeruginosa	437392	NA	Clinical	NA	NZ_CP029096.1
pPABL048	56.58	1.54E+05	6.20E+05	70	0	98.54	P. aeruginosa	414954	2001	Clinical	USA	NZ_CP039294.1
Unnamed2-AR_0356	57.14	1.53E+05	6.49E+05	73	0	98.26	P. aeruginosa	438531	NA	Clinical	USA	NZ_CP027170.1
p727-IMP	56.39	1.25E+05	7.35E+05	75	0	99.61	P. aeruginosa	430173	NA	Clinical	China	MF344568.1
Unnamed3-AR441	57.14	1.22E+05	6.49E+05	73	0	98.16	P. aeruginosa	438529	NA	Clinical	USA	NZ_CP029094.1
pJB37	57.20	1.21E+05	6.81E+05	74	0	98.76	P. aeruginosa	464804	2008	Clinical	Portugal	KY494864.1
pBM908	56.86	1.15E+05	7.37E+05	74	0	100	P. aeruginosa	395774	2018	Clinical	China	CP040126.1
pRBL16	55.57	1.09E+05	6.31E+05	71	0	98.80	P. citronellolis	370338	2015	Sludge	China	NZ_CP015879.1
pR31014-IMP	56.37	8.75E+04	6.93E+05	68	0	99.99	P. aeruginosa	374000	NA	Clinical	China	MF344571.1
pA681-IMP	56.35	8.15E+04	7.17E+05	71	0	98.92	P. aeruginosa	397519	NA	Clinical	China	MF344570.1
pSY153-MDR	56.56	7.71E+04	9.09E+05	83	0	99.38	P. putida	468170	2012	Clinical	China	KY883660.1
pTTS12	57.85	75188	6.35E+05	71	0	98.42	P. putida	583900	1989	Soil	Netherlands	NZ_CP009975.1
plasmid1-RW109	58.09	74550	6.35E+05	71	0	99.22	P. aeruginosa	555265	NA	Industrial	NA	LT969519.1

a NA, no data available.

To further investigate and understand the relationships between pPAG5 and related megaplasmids, we constructed a phylogenetic tree based on the concatenated alignment of the high-quality single-nucleotide polymorphisms (SNPs) using the CSI Phylogeny 1.4 software (21). A close phylogenetic relationship was found among pPAG5, pOZ176, and pJB37, from the P. aeruginosa PAG5, P. aeruginosa PA96 and P. aeruginosa FFUP_PS_37 clinical isolates, respectively (Fig. 4). All three of these megaplasmids were isolated from clinical P. aeruginosa isolates, and they belong to the same IncP-2 group. Comparative analysis of the 18 complete megaplasmid sequences using BLAST Ring Image Generator (BRIG) revealed that the pPAG5 nucleotide sequence is similar to those of 17 other related megaplasmids (Fig. 2). Theses plasmids shared similar IncP-2 backbones and key traits, but there were several DNA fragment differences between pPAG5 and the 17 megaplasmids (Fig. 2). In particular, the ∼13-kb variable region 1 (VR1; bp 159,544 to 171,526) and the ∼41-kb variable region 2 (VR2; bp 11,910 to 51,201) of pPAG5 were absent from any of the other megaplasmids. A BLASTn search of VR1 showed that it was similar (82% coverage and 100% identity) to the IMP-harboring p420352-IMP plasmid (GenBank accession number MN961670.1) isolated from P. putida strain 420352. The VR1 fragment was also similar (75% coverage and 96.12% identity) to the AG1 chromosome of a P. aeruginosa strain that was recently described in the first report of a P. aeruginosa isolate carrying both *bla*_VIM-2_ and *bla*_IMP-18_ resistance genes (22). It is noteworthy that VR2 was highly similar to the PAG5 host strain of megaplasmid pPAG5 (94% query coverage and 99.93% nucleotide similarity). Annotation of the unique VR1 and VR2 regions suggested that they had not contributed to any resistance genes. However, the majority of their genes were responsible for encoding type I restriction-modification system endonuclease, which is closely associated with defense against invading foreign DNA and maintaining the integrity of the host genome (23).

## DISCUSSION

In this study, a hybrid approach combining Nanopore and Illumina sequencing was applied to identify and characterize a megaplasmid in a clinical P. aeruginosa isolate. To the best of our knowledge, plasmid pPAG5 is the largest MDR plasmid ever sequenced in the Pseudomonas genus. *In silico* analysis of the sequence features showed that pPAG5 was closely related to IncP-2 plasmids. It possesses substantial numbers of diverse mobile elements, such as insertion sequences (ISs), integrons, and transposons. These mobile elements are considered important vehicles for transmitting resistance genes and promote the exchange and rearrangement of genetic information (2). Both ends of the VR1 and VR2 variable regions are ISs in pPAG5; VR1 is flanked by *recA*-like and IS*6100*, while VR2 is bordered by IS*1411* and IS*Pa1382*. The findings revealed that the variable regions were likely captured by homologous recombination.

Two MDR regions in pPAG5 also harbor transposases and integrases, indicating a key role for these mobile genetic elements in the acquisition of resistance and in evolution (12). This suggests that complex rearrangement and homologous recombination events likely occurred during the evolution of pPAG5 (15, 18, 24). Recent analysis showed that members of the megaplasmid family contained diverse resistance elements to form AMR regions via homologous recombination, and some of the resistance genes carried trace back to the late 1970s (2).

Megaplasmids may harbor accessory modules that provide adaptive advantages or broaden the host strain response spectrum. Such advantages include virulence factors, antibiotic resistance, and heavy metal resistance (2). The IncP-2 megaplasmids, such as pPAG5, pOZ176, pJB37, and pBM413, have a common core genetic backbone but different AMR gene profiles, suggesting that the backbone was formed first and then collected diverse cassettes of AMR genes during transfer to different host strains (5, 6, 24). The two larger AMR regions identified in pPAG5 are highly similar to the MDR regions in megaplasmid pSY153 in a P. putida isolate from a clinical environment in China (15). The MDR-1 genes also have a best match to the MDR region in plasmid pPA1819 in P. aeruginosa strain 14.1819 isolated from a clinical environment in France (14). This difference of the plasmid backbone suggests that the resistance genes have been assembled independently of the backbone and that horizontal gene transfer plays an important role in the dissemination of resistance genes in the clinical setting.

Some members of the IncP-2 megaplasmid family are considered conjugative plasmids (2, 5, 6), but they differ in their transfer genes; for example, pOZ176 carries *traF*, *traG*, *virD2*, and *trbBCDEJLFGI* genes, while pBT2436 carries *traGBV*, *dnaG*, and type IV pilus-related/type II secretion genes. Other members are nonconjugative because transfer genes are absent or disrupted (3, 15, 25). We found that the *virB4* gene and *traG* gene are encoded on pPAG5. The VirB-like protein, VirD4, and TraG-like protein interact with the DNA substrate and couple it to the secretion pore via mating pair formation (19, 26). Additionally, the *traI* gene is located upstream from the type IV secretion system (T4SS) cluster. This suggests that the *traI* gene may serve as a relaxase and is involved in initiating DNA transfer (19). Comparative analysis of the transfer region of pPAG5 revealed that it contains a P-type T4SS with all genes required for transfer, which forms a conjugative pilus and mediates mating pair stabilization (27). Importantly, conjugation experiments revealed that pPAG5 can be successfully self-transmissible into recipient strains.

In conclusion, we report a conjugative transferable plasmid, pPAG5, from a P. aeruginosa isolate in a hospital environment. It belongs to the IncP-2 group plasmids and carries two large MDR regions. It poses a serious threat, especially for controlling nosocomial infections. To defuse this threat, it is crucial to prevent the rapid transmission of MDR plasmids into our health care systems. A better understanding of the problem of how resistance plasmids evolve in hospitals and the environment is a key to controlling the global threat of antibiotic resistance.

## MATERIALS AND METHODS

### Bacterial strains and plasmids used in this study.

The strains and plasmids used in this study are listed in Table 3. P. aeruginosa isolate PAG5 exhibited an MDR profile, as previously described (9). Escherichia coli strains were maintained in Luria Bertani (LB) broth or LB agar at 37°C. For solid cultivation, 1.5% (wt/vol) agar was added to LB. All P. aeruginosa strains were grown in LB or Pseudomonas isolation agar at 37°C.

**TABLE 3:** Strains and plasmids used in this study

Strain or plasmid	Relevant characteristics or function	Source
Strains		
P. aeruginosa PAG5	A multidrug-resistant clinical isolate from a urine sample	This study
PAO1-*lux*	A derivative of P. aeruginosa PAO1, carrying *luxCDABE* genes driven by the *modA* promoter	This study
E. coli DH5α-*lux*	E. coli DH5α containing plasmid pMS402 with a promoterless *luxABCDE* reporter gene driven by the *modA* promoter	This study
Plasmids		
pMS402	*lux-*based promoter reporter plasmid; Km^r^ Tp^r^	This study
pPAG5	Natural 513-kbp plasmid of P. aeruginosa PAG5, carrying diverse resistance genes	This study

### Antimicrobial susceptibility determination.

The isolates were assessed for MICs of the tested antibiotics by the broth microdilution method according to the 2021 Clinical and Laboratory Standards Institute (CLSI) guidelines (28). The antimicrobial agents tested included amikacin, aztreonam, ceftazidime, ciprofloxacin, colistin, cefepime, gentamicin, imipenem, levofloxacin, meropenem, piperacillin, and piperacillin-tazobactam.

### Construction of recipient strains.

The broad antibiotic resistance conferred by pPAG5 complicates the plasmid transmission assay because selecting, enumerating, and isolating transconjugants usually requires a selective marker carried by recipient strains. Therefore, engineered bioluminescent reporter recipient strains of P. aeruginosa PAO1 and E. coli DH5α, carrying *luxABCDE* (driven by the *modA* promoter of PAO1), were constructed. To construct the *lux* reporter strains, the plasmid pMS402, with a promoterless *luxABCDE* reporter gene cluster, was used as previously described (29). The *modA* promoter region was amplified by PCR from genomic DNA of PAO1 and cloned into the BamHI-XhoI sites upstream from the *lux* genes in pMS402, resulting in a *modA*-*lux* fusion plasmid named pKD-*modA*. The promoter region was confirmed by DNA sequencing. Apart from the plasmid-based *lux* reporter system, an integration plasmid, CTX 6.1, derived from the plasmid mini-CTX-*lux*, was used to construct the chromosomal fusion reporter (30). The pMS402 fragment containing the MCS kanamycin resistance marker and *modA*-*lux* reporter cassette was ligated into integration plasmid CTX 6.1 with the PacI site (31). The new recombinant plasmid was than transformed into PAO1 by electroporation and integrated into the *attB* chromosome site of PAO1 as previously described (32). This yielded a bioluminescent strain named PAO1-*lux* (Fig. 3). pKD-*modA* was transformed into E. coli DH5α by electroporation, yielding E. coli strain DH5α-*lux*.

### Conjugation experiments.

Conjugation experiments were performed as previously described, with a slight modification (33). The donor strain was the PAG5 clinical isolate. PAO1-*lux* and E. coli DH5α-*lux* played roles as recipient strains. Overnight donor and recipient bacteria were combined in a 10:1 ratio in 1 mL LB broth and then cocultured using filters on solid LB agar at 37°C without agitation for 12 h. Then, the mixture was resuspended in LB broth and plated onto Mueller-Hinton agar plates supplemented with gentamicin (100 µg/mL). After incubation for 24 h at 37°C, growing colonies with bioluminescence were selected as transconjugants. Plasmid acquisition in the transconjugants was further confirmed by antibiotic susceptibility tests and detection of the gentamicin resistance gene *armA* of the plasmid by PCR.

### Plasmid analysis.

Plasmid pPAG5 was annotated with the NCBI Prokaryotic Genome Annotation Pipeline (PGAP, version 4.9) (https://www.ncbi.nlm.nih.gov/genome/annotation_prok/) and the RAST server (version 2.0) (http://rast.nmpdr.org/). This was followed by manual review through BLASTp search. Function of genes were annotated via EggNOG mapper (version 4.5.1) (34). The incompatibility type of the plasmid pPAG5 was determined by using the Plasmid Finder (version 2.1) (https://cge.cbs.dtu.dk/services/PlasmidFinder/) and Plasmid MLST (https://pubmlst.org/plasmid/) databases. The resistance determinants were identified using ResFinder (version 3.2) (https://cge.cbs.dtu.dk/services/ResFinder/) and RGI software from the CARD database (https://card.mcmaster.ca/). Additional software, including Transposon Registry (35), Isfinder (36), and INTEGRALL (version 1.2) (37), was used to identify mobile genetic elements.

### Comparative analysis of pPAG5 and related megaplasmids.

Complete pPAG5-related megaplasmid sequences deposited in GenBank were identified through BLASTn searches against the nonredundant GenBank database (up to October 2019), using pPAG5 as a query sequence. The maximum scores reflect complete homologous sequences. Pairwise comparisons of pPAG5 and related megaplasmids were generated using BLAST Ring Image Generator (BRIG; version 0.95) (38). Easyfig was used for comparative analysis of MDR regions. The phylogenetic trees were constructed from single-nucleotide polymorphism (SNP) concatemers using the CSI Phylogeny 1.4 software (21). The phylogenetic trees and AMR gene contents were visualized and edited with the iTOL tool (version 4.3.3) (39).

### Data availability.

The complete sequence of plasmid pPAG5 has been submitted to GenBank under accession number CP045003.
